# Recent poliovirus outbreaks and vaccination: A perspective

**DOI:** 10.1016/j.amsu.2022.104970

**Published:** 2022-11-18

**Authors:** Yashendra Sethi, Nirja Kaka, Hitesh Chopra, Navidha Aggarwal, Sonia Arora, Talha Bin Emran

**Affiliations:** Government Doon Medical College, Dehradun, Uttarakhand, India; GMERS Medical College, Himmatnagar, Gujarat, India; Chitkara College of Pharmacy, Chitkara University, Punjab, India; MM College of Pharmacy, Maharishi Markandeshwar (Deemed to Be University), Mullana, 133207, Ambala, Haryana, India; Chandigarh College of Pharmacy, Chandigarh Group of Colleges, Landran, Punjab, India; Department of Pharmacy, BGC Trust University Bangladesh, Chittagong, 4381, Bangladesh; Department of Pharmacy, Faculty of Allied Health Sciences, Daffodil International University, Dhaka, 1207, Bangladesh

**Keywords:** Poliomyelitis, Poliovirus, Immunodeficiency, Public health emergency, Vaccine resistance

Poliomyelitis is an enteroviral disease, caused by “Poliovirus” a non-enveloped RNA virus, belonging to the Picornaviridae family. Three serotypes of Poliovirus (PV1, PV2, and PV3) with minimal heterotypic immunity are known [1]. It is transmitted through the feco-oral route and primarily affects children less than 5 years old. Up to three-fourths of poliovirus infections are asymptomatic; however, around 24% experience constitutional symptoms such as low-grade fever and sore throat [2]. Around 1% of cases face CNS spread causing transient or permanent acute flaccid paralysis (AFP), termed Poliomyelitis [3,4]. Polio has been a cause of debilitating outbreaks and high morbidity, and mortality, but owing to global eradication programs, vaccination campaigns, and improved health education, infection is either eliminated or on the verge of being eliminated from the world. The widespread vaccination have decreased the incidence of poliomyelitis by 99% over the past four decades [4]. The wild poliovirus types 2 and 3 were declared eradicated in 2015 and 2019, respectively. However, the transmission of wild poliovirus type 1 (WPV1) has been detected only in Afghanistan and Pakistan [5]. Vaccine-derived poliovirus cases are reported periodically from other parts of the world, especially in Asia and Africa.

The attenuated virus can rarely mutate, causing vaccine-derived poliovirus (VDPV) or vaccine-associated paralytic polio (VAPP). VDPV is genetically divergent having prolonged replication or transmission, however, VAPP is an adverse event following exposure (AEFI) to oral polio vaccine (OPV). VDPVs are broadly divided into three major types: (1) circulating VDPVs (cVDPVs) seen in settings of low OPV coverage, (2) immunodeficiency-associated VDPVs (iVDPVs) seen in population-clusters with primary immunodeficiencies, and (3) ambiguous VDPVs (aVDPVs). PV2 is attributed to cause most VDPVs and hence WHO is working to shift from trivalent OPV to bivalent OPV containing only poliovirus types 1 and 3, before transition to Injectable viral vaccines with no such risk [3].

Recent times have seen outbreaks in the United States, UK and Africa except the known clusters in Afganistan and Pakistan. The Centers for Disease Control and Prevention (CDC) was notified in July 2022 of a case of polio caused by vaccine-derived poliovirus type 2 (VDPV2) in an unvaccinated individual from Rockland County, New York. Similarly, a case was reported on September 13, 2022. The virus has been isolated from several wastewater samples from communities near the patient's residence and thus meets the World Health Organization (WHO criteria)'s for circulating vaccine-derived poliovirus (cVDPV) [6].

Similar reports have also been reported from other parts of the world, on 8 July 2022, a case of vaccine-derived poliovirus type 2 (cVDPV2) was reported to WHO through the Global Polio Laboratory Network from Algeria (GPLN) [7]. Pasteur Institute of Algeria and Pasteur Institute of Paris both verified the presence of cVDPV2 in stool samples. The isolated virus is genetically related to a virus previously identified in Kano, Nigeria. The youngster had never been vaccinated against polio and had never been outside of the Tamanrasset province. This is the first incidence of cVDPV2 in Algeria, where, according to WHO-UNICEF forecasts for 2021, Pol3 (third dose of polio-containing vaccine) coverage was 91% and IPV1 (one dose of the inactivated polio vaccine) coverage was 93% [8,9].

VDPVs may create epidemics in areas with poor vaccination rates. Moreover, persons with specific immunodeficiency conditions may shed the virus for extended periods of time, during which the virus can continue to evolve and infect an unprotected individual. Since OPV has not been used in the United States since 2000 and IPV vaccination coverage is high, it is doubtful that any VDPV would gain widespread usage in the United States [8].

These have pushed the alarm for the longest ongoing global public health emergency of Polio as the cases visible may just be the ‘tip of the iceberg’ and for one case reported there might be hundreds who got infected [10]. It has been observed that the outbreaks in these regions, especially the US have been more common in areas of altered vaccine coverage [11]. The CDC highlighted that the most vulnerable people to polio infection are those who are not vaccinated or incompletely vaccinated. The vaccines in the case of Polio have been a great success story with OPV aiding the herd immunity to combat pandemics and replacement with IPV covering individual protection and preventing VDPV. Despite the success of vaccines, the vaccine resistance among some clusters of the population has created a vulnerability that warrants attention. Eradication of a disease cannot be thought of unless we cover all vulnerable groups. The recent COVID-19 pandemic and the lockdowns have just added to the trouble forcing troubles in vaccination programs [12].

The polio vaccine protects against both naturally occurring and vaccine-derived polioviruses. Continuing to attain high IPV vaccination coverage in the United States is the greatest approach to maintaining the country's polio-free status and preventing the virus' importation. Furthermore, access to clean water, excellent hand hygiene, modern sewage systems and wastewater management minimize the spread of pathogens, such as poliovirus. Polio eradication continues to be a CDC global health priority in order to safeguard children from this avoidable illness. There is a persistently high risk of worldwide transmission of cVDPV2 owing to poor immunity, monitoring inadequacies, and massive population migrations. Reduced vaccination rates due to the current COVID-19 epidemic have also aggravated the risk.

More than 20 countries have reported cVDPV outbreaks and WPV is still endemic in two countries which is always posing the world at risk of another major pandemic. It is crucial that all countries, especially those with frequent travel and contact with polio-affected regions, increase surveillance for AFP and initiate the planned expansion of environmental surveillance in order to detect any new virus importation facilitating a rapid response. WHO's international travel and health advises polio vaccination for all travelers to polio-affected regions. Residents (and visitors for more than 4 weeks) from infected regions should take a booster dose of oral polio vaccine (OPV) or inactivated polio vaccine (IPV) within four to twelve months prior to departure [6].

The oral polio vaccine (OPV) consists of a live attenuated virus that carries the risk of viral mutation, neuro-virulence reversal, and reactivation causing vaccine-associated paralytic poliomyelitis (VAPP). Type 3 strain has been more strongly linked to the VAPP than other strains. Immunodeficient patients should not be given the OPV due to the higher risk of re-activation. To prevent the re-emergence of poliomyelitis due to VAPP, OPV should be used with utmost caution and only in areas with higher prevalence [13].

We recommend improving health education through behavior change communication (BCC) and Focused group discussions (FGD) by trained workers to improve vaccine acceptability and coverage in all pockets. We also advocate the idea of strengthening both AFP surveillance and environmental surveillance. A country-specific, precise, and individualised strategy is required to eradicate WPV and cVDPV simultaneously [9]. Furthermore, helping the implementation of IPVs in all national vaccination programs globally can substantially help reduce the AEFI of VAPP and VDPV [14].

## Ethical approval

Not applicable.

## Sources of funding

No funding.

## Author contributions

**Yashendra Sethi:** Conceptualization, Data curation, Writing-Original draft preparation, Writing- Reviewing and Editing. **Nirja Kaka:** Conceptualization, Data curation, Writing-Original draft preparation, Writing- Reviewing and Editing. **Hitesh Chopra:** Data curation, Writing-Original draft preparation, Writing- Reviewing and Editing. **Navidha Aggarwal:** Data curation, Writing-Original draft preparation, Writing- Reviewing and Editing. **Sonia Arora:** Writing-Reviewing and Editing, Visualization. **Talha Bin Emran:** Writing- Reviewing and Editing, Visualization, Supervision.

## Declaration of competing interest

Authors declare that they have no conflicts of interest.

## Provenance and peer review

Not commissioned, internally peer-reviewed.

## Data statement

No specific data collected for the above manuscript.
